# Sex-related differences in spontaneous intracerebral hemorrhage outcomes: A prognostic study based on 111,112 medical records

**DOI:** 10.3389/fneur.2022.957132

**Published:** 2022-09-23

**Authors:** Jieyi Zhao, Tao Zhang, Hongli Wan, Yang Yu, Jin Wen, Xiaoyu Wang

**Affiliations:** ^1^Department of Neurosurgery, West China Hospital, Sichuan University, Chengdu, China; ^2^West China School of Public Health, Sichuan University, Chengdu, China; ^3^Institute of Hospital Management, West China Hospital, Sichuan University, Chengdu, China

**Keywords:** stroke, outcome, factor, sex, data

## Abstract

**Objective:**

To identify sex-related differences in the outcome of hospitalized patients with spontaneous intracerebral hemorrhage (SICH), and to identify potential causal pathways between sex and SICH outcome.

**Methods:**

A total of 111,112 medical records of in-hospital patients with SICH were collected. Data- and expert-driven techniques were applied, such as a multivariate logistic regression model and causal mediation analysis. These analyses were used to determine the confounders and mediators, estimate the true effect of sex on the SICH outcome, and estimate the average causal mediation effect for each mediator.

**Results:**

(1) Failure (disability or death) rates in women with SICH were significantly lower than in men with SICH. On the day of discharge, the odds ratio (*OR*) of failure between women and men was 0.9137 [95% confidence interval (CI), 0.8879–0.9402], while the odds ratio at 90 days post-discharge was 0.9353 (95% confidence interval, 0.9121–0.9591). (2) The sex-related difference in SICH outcome decreased with increasing age and disappeared after 75 years. (3) Deep coma, brainstem hemorrhage, and an infratentorial hemorrhage volume of >10 ml accounted for 62.76% (*p* < 0.001), 33.46% (*p* < 0.001), and 11.56% (*p* < 0.001) of the overall effect on the day of discharge, and for 52.28% (*p* < 0.001), 27.65% (*p* < 0.001), and 10.86% (*p* < 0.001) of the overall effect at the 90-day post-discharge.

**Conclusion:**

Men have a higher failure risk than women, which may be partially mediated by a higher risk for deep coma, brainstem hemorrhage, and an infratentorial hemorrhage volume of >10 ml. Future work should explore the biological mechanisms underlying this difference.

## Introduction

Sex differences in nervous system disease outcomes have received increasing attention, such as recent work in patients with stroke and cerebral amyloid angiopathy (1). However, the effects of sex-related differences in outcomes of spontaneous intracerebral hemorrhage (SICH) remain unclear; this knowledge could facilitate an understanding of the mechanisms underlying SICH and the development of new treatment and prevention approaches (2–5). Only a few studies have explored the effect of sex on SICH prognosis, and these have yielded inconsistent findings (3, 6–9). Such inconsistencies could be explained by limitations, such as small sample sizes and the presence of confounding bias (2, 7, 10–13). Furthermore, the causal pathways underlying sex-related outcomes remain unknown. These gaps in knowledge impede the development of preclinical research models and therapies for SICH; further studies with larger sample sizes and more causal considerations are thus necessary. To this aim, the present study used the largest number of in-hospital medical records to date (i.e., 111,000) to investigate the effect of sex on SICH outcomes. Moreover, we put forward some possible causal pathways between sex and SICH, which could be further examined in future research.

## Materials and methods

### Study design and participants

This study is a retrospective cross-sectional study of consecutive patients referred to the governmental hospital in the Sichuan province. Data- and expert-driven techniques were applied, including a multivariate logistic regression model and causal mediation analysis.

The data were collected from the database of the Comprehensive Data Collection and Decision Support System for health statistics of Sichuan Province, which has a jurisdiction area of 485,000 km^2^ and a population of about 83 million. This database was constructed by the Sichuan government on 1 January 2017 and includes information about all SICH hospital admissions to date, including medical records from all general hospitals and community hospitals in Sichuan. The database contains clinical data, such as demographic characteristics, diagnoses, comorbidity, treatment, and the medical record home page.

Patients were identified by the International Classification of Diseases, Tenth Revision, Clinical Modification, and only the patients with nontraumatic intracerebral hemorrhage (I61) were included in the study. To avoid measuring the effect of other secondary causes of hemorrhage, such as aneurysm, vascular malformations, and coagulopathy, patients with an intracranial tumor, aneurysm, or other vascular malformation presumed to be the cause of the hemorrhage and patients with hemorrhagic conversion of acute brain infarction and secondary ICH were excluded, even though they were in the I61 group.

Detailed information on patient demographics (age, sex, and ethnicity), brain imaging, stroke severity, diagnosis, treatment, complications, comorbidities, instant discharge outcome, and 90-day outcome was collected. Brain imaging included location [lobar (predominantly cortical or subcortical white matter), depth (predominantly basal ganglia, internal capsule, or periventricular white matter), cerebellum, brainstem, and ventricle]. The time hospitalized variable was the hospitalization history of patients prior to the current SICH hospitalization. Stroke severity was described as severe coma, moderate coma, and minor coma, according to the Glasgow Coma Scale (GCS; sober, GCS score = 15; shallow coma, GCS score = 12–14; mediate coma, GCS score = 9–11; and deep coma, GCS score ≤8). Complications and comorbidities included hypertension, diabetes, and infections. The SICH outcome was dichotomized as “success” or “failure.” A successful outcome was defined as a score of 2 or more on the Glasgow Outcome Scale (GOS), and failure was defined as discharge to a hospice or a GOS score of 1. The SICH outcome was measured two times, one time on the day of discharge and the other at 90 days post-discharge. The first outcome was obtained from the database, and the second one was verified through the Ministry of Civil Affairs through personal identification numbers.

Furthermore, since the unstructured variables (such as diagnosis) in this study were all in sentence forms, we carried out natural language processing to transform them into a structured form for further analysis. We first pre-processed the unstructured data, including word segmentation and the removal of stop words. Then, the Tagged Document in the Gensim package was used to wrap the input sentence and change it to the input sample format required by Doc2vec. After that, we loaded the Doc2vec model with a window size of three and began model training. Finally, the unstructured data were transformed into numeric codes.

### Standard protocol approvals, registrations, and patient consent

The study was approved by the West China Hospital's institutional review board, and informed consent was obtained from all participants.

### Statistical analysis

To explore the sex differences in SICH outcomes, two types of variables (i.e., confounders and mediators) needed to be fully considered. Confounders can cause spurious associations that conceal the true effect of sex on SICH outcomes and were therefore adjusted before the analysis. On the other hand, mediators form part of the causal pathway between sex and SICH outcome. Given that there might be multiple causal pathways and corresponding mediators, we assessed the extent to which the effect of sex on the SICH outcomes was mediated through a particular pathway and mediator. Therefore, the statistical analysis was carried out in the following three steps:

(1) Determination of confounders and mediators using the mixture-driven method.

By definition, both confounders and mediators are correlated to the exposure and outcome, but they differ in that confounders are not part of the causal pathway and mediators are. Given this distinction, we used a mixture of data- and expert-driven methods to identify the confounders and mediators. Specifically, the data-driven method included association analysis to first select candidate variables that were correlated with both sex and SICH outcomes. Then, for every candidate variable, three experienced neurologists were asked to decide whether it was a confounder or mediator according to the current research and their clinical experience. As a result, sets of confounders and mediators were defined for further analysis.

(2) The control of confounders using a multivariate logistic regression model.

This study built two multivariate logistic regression models that contained two observation time points—the day of discharge and 90 days after discharge. Other than sex, all confounders were included in the regression models to control for confounding bias.

(3) Pathway exploration using causal mediation analysis.

After determining the effect of sex on the SICH outcome, we examined the underlying causal pathway(s) between sex and SICH outcome that could explain the observed effect from a mechanical point of view. However, identifying the precise mechanisms underlying this association was beyond the scope of this study because the biological and pathological data were unavailable. That said, using a causal mediation analysis, we were able to at least provide some clues to pathway construction. The goal of the causal mediation analysis was to assess the direct and indirect effects of sex on SICH outcomes and estimate the average causal mediation effect for each mediator. After this, each mediator was ranked by its corresponding average causal mediation effect such that their relative importance could be established. This relative importance points to the most likely causal pathways between sex and SICH outcome, which provides a platform for future research.

In addition, we identified variables that were unevenly distributed across the sexes and may be associated with clinical outcomes. Using correlation analysis and expert consultation, confounding factors and mediating variables were defined. The influence of confounding factors on the association between sex and outcome variables was corrected by using multivariate regression analysis, which ensured that this imbalance would not affect the results.

All analyses were performed in R 3.5.0, using R packages {stats} (4, 14) and {mediation} (9), which were downloaded from the Comprehensive R Archive Network at http://cran.r-project.org/ and installed in advance. The default significance level (α) was 0.05 unless otherwise specified.

## Results

From 1 January 2017 to 30 June 2019, a total of 117,227 patients with SICH were screened and 111,112 met the inclusion criteria [68,326 (58.3%) women and 42,786 (41.7%) men]. Table 1 shows the explanations for each variable.

**Table 1:** Explanations for each variable.

**Variable label**	**Value**	**Male (*N* = 68,326)**	**Female (*N =* 42,786)**	**χ^2^**	***P***
Age group	(40, 54)	15,986 (23.40%)	8,570 (20.03%)	663.97	< 0.001
	(55, 64)	15,216 (22.27%)	8,417 (19.67%)		
	(65, 74)	21,412 (31.34%)	13,460 (31.46%)		
	(75, 84)	12,806 (18.74%)	9,501 (22.21%)		
	>84	2,906 (4.25%)	2,838 (6.63%)		
Ethnicity	Han	66,958 (98.00%)	42,022 (98.21%)	6.44	0.011
	Non-han	1,368 (2.00%)	764 (1.79%)		
Times of in-hospital	< 2	67,153 (98.28%)	42,493 (99.32%)	214.41	< 0.001
	≥2	1,173 (1.72%)	293 (0.68%)		
Deep Coma	No	60,865 (89.08%)	38,594 (90.20%)	35.17	< 0.001
	Yes	7,461 (10.92%)	4,192 (9.80%)		
Location	Deep	45,188 (66.14%)	28,626 (66.91%)	311.80	< 0.001
	Lobar	11,877 (17.38%)	7,588 (17.73%)		
	Brainstem	5,731 (8.39%)	2,482 (5.80%)		
	Cerebellum	3,584 (5.24%)	2,731 (6.38%)		
	Ventricle	1,946 (2.85%)	1,359 (3.18%)		
Infratentorial	< 10	58,980 (97.55%)	36,981 (98.07%)	28.99	< 0.001
	≥10	1,484 (2.45%)	727 (1.93%)		
Hypertension	No	52,304 (76.55%)	34,185 (79.90%)	170.67	< 0.001
	Yes	16,022 (23.45%)	8,601 (20.10%)		
Diabetes	No	68,114 (99.69%)	42,599 (99.56%)	11.47	< 0.001
	Yes	212 (0.31%)	187 (0.44%)		
Operation	No	59,067 (86.45%)	37,462 (87.56%)	28.23	< 0.001
	Yes	9,259 (13.55%)	5,324 (12.44%)		
Infection	No	44,723 (65.46%)	29,245 (68.35%)	99.06	< 0.001
	Yes	23,603 (34.54%)	13,541 (31.65%)		

### The crude effect of sex on SICH prognosis

As shown in Table 2, on both the day of discharge and the 90-day post-discharge, female patients had a lower failure risk than male patients. Furthermore, this phenomenon was found for all age groups overall as well as the age subgroups of 40–54, 55–64, and 65–74 years.

**Table 2:** The univariate estimation of sexual effect on prognosis outcomes.

**Prognosis outcome**	**Stratification**	**Regression coefficients**	**SE**	**OR**	**95% CI for OR**
The instant discharge outcome (1. Success; 2. Failure)	**All age groups**	**−0.0783**	**0.0145**	**0.9247**	**(0.8988, 0.9513)**
	**(40, 54)**	**−0.2330**	**0.0330**	**0.7922**	**(0.7425, 0.8451)**
	**(55, 64)**	**−0.2527**	**0.0337**	**0.7767**	**(0.7271, 0.8297)**
	**(65, 74)**	**−0.0530**	**0.0260**	**0.9484**	**(0.9013, 0.9980)**
	(75, 84)	0.0125	0.0304	1.0126	(0.9540, 1.0747)
	>84	0.0420	0.0558	1.0429	(0.9349, 1.1634)
The 90-day post discharge outcome (1. Success; 2. Failure)	**All age groups**	**−0.0663**	**0.0127**	**0.9359**	**(0.9128, 0.9594)**
	**(40,54)**	**−0.1425**	**0.0279**	**0.8672**	**(0.8210, 0.9159)**
	**(55, 64)**	**−0.1420**	**0.0284**	**0.8676**	**(0.8206, 0.9173)**
	**(65, 74)**	**−0.0541**	**0.0228**	**0.9473**	**(0.9059, 0.9906)**
	(75, 84)	**–**0.0113	0.0277	0.9888	(0.9365, 1.0439)
	>84	0.0168	0.0532	1.0169	(0.9162, 1.1287)

The bold values indicate the group that female patients had a lower failure risk than male patients.

### Determination of confounders and mediators

The data-driven method for the determination of confounders and mediators included a correlation analysis between candidate variables and sex groups, a univariate logistic regression analysis that examined the effect of each candidate variable on the SICH outcome on the day of discharge, and a univariate logistic regression analysis that examined the effect of each candidate variable on the SICH outcome at the 90-day post-discharge time point. The correlation analysis revealed that the age group (χ^2^ = 663.97, ν = 4; *p* < 0.001), number of in-hospital stays (χ^2^ = 214.41, ν = 1; *p* < 0.001), operation (χ^2^ = 28.23, ν = 1; *p* < 0.001), infection (χ^2^ = 99.06, ν = 1; *p* < 0.001), deep coma (χ^2^ = 35.17, ν = 1; *p* < 0.001), location (χ^2^ = 311.80, ν = 4; *p* < 0.001), supratentorial hemorrhage volume of >30 ml (χ^2^ = 27.38, ν = 1; *p* < 0.001), and infratentorial hemorrhage volume of >10 ml (χ^2^ = 28.99, ν = 1; *p* < 0.001) were significantly correlated with sex. Tables 3, 4 summarize the results of the univariate logistic regression models, which revealed that these above-mentioned variables were significantly associated with the SICH outcome both on the day of discharge and at the 90-day post-discharge time point. After discussions with experienced neurologists, the age group, number of in-hospital stays, operation, and infection were defined as confounders, and deep coma, location, supratentorial hemorrhage volume of >30 ml, and infratentorial hemorrhage volume of >10 ml were defined as mediators. These determinations are described in more detail in the Discussion section.

**Table 3:** The effect of each candidate variable on the spontaneous intracerebral hemorrhage (SICH) outcome on the day of discharge.

**Variables**	**Estimated coefficients**	**SE**	***t*-value**	***P***
Age group	0.1200	0.0060	20.08	< 0.001
Ethnicity	**–**0.0338	0.0515	**–**0.66	0.512
Times of in hospital	0.3513	0.0569	6.17	< 0.001
Deep Coma	3.3687	0.0277	121.70	< 0.001
**Location**				
Deep	—*	—*	—*	—*
Lobar	0.2381	0.0256	9.32	< 0.001
Brainstem	0.9696	0.0241	40.31	< 0.001
Cerebellum	0.1360	0.0307	4.42	< 0.001
Ventricle	0.4300	0.0390	11.02	< 0.001
Supratentorial	0.7974	0.0183	43.62	< 0.001
Infratentorial	1.9367	0.0461	41.99	< 0.001
Hypertension	0.0253	0.0168	1.50	0.133
Diabetes	0.0495	0.1157	0.43	0.669
Operation	0.0755	0.0205	3.69	< 0.001
Infection	0.2199	0.0146	15.04	< 0.001

^*^Comparison group.

**Table 4:** The effect of each candidate variable on the SICH outcome at the 90-day post-discharge.

**Variables**	**Estimated coefficients**	**SE**	***t*-value**	***P***
Age group	0.0455	0.0053	8.64	< 0.001
Ethnicity	**–**0.0569	0.0454	**–**1.25	0.210
Times of in-hospital	0.1412	0.0534	2.64	0.00819
Deep Coma	2.7824	0.0297	93.67	< 0.001
**Location**				
Deep	—*	—*	—*	—*
Lobar	0.1887	0.0227	8.31	< 0.001
Brainstem	0.6648	0.0234	28.43	< 0.001
Cerebellum	0.0545	0.0271	2.01	0.044
Ventricle	0.3116	0.0359	8.68	< 0.001
Supratentorial	0.7591	0.0174	43.73	< 0.001
Infratentorial	1.6604	0.0501	33.10	< 0.001
Hypertension	0.0006	0.0149	0.04	0.966
Diabetes	0.0913	0.1023	0.89	0.373
Operation	0.2492	0.0180	13.85	< 0.001
Infection	0.2056	0.013	15.79	< 0.001

^*^Comparison group.

### The effect of sex on SICH outcomes after confounder adjustment

In the next step, we added confounders as covariates into the logistic regression model of sex on SICH outcome. As shown in Table 5, female patients had lower risks of failure both on the day of discharge and at 90-day post-discharge than did male patients [day of discharge: odds ratio (OR) = 0.91, 95% confidence interval (CI), 0.89–0.94; and 90-day post-discharge: odds ratio = 0.94, 95% confidence interval, 0.91–0.96].

**Table 5:** The multivariate estimation of sex on the SICH outcome.

**Variables**	**The day of discharge**		**The 90-day post-discharge**	
	**OR**	**95% CI**	**OR**	**95% CI**
Sex	0.9137	(0.8879, 0.9402)	0.9353	(0.9121, 0.9591)
Age group	1.1272	(1.1138, 1.1407)	1.0516	(1.0405, 1.0628)
Times of in-hospital	1.4002	(1.2519, 1.5660)	1.1565	(1.0412, 1.2846)
Operation	1.0671	(1.0234, 1.1125)	1.2433	(1.1986, 1.2898)
Infection	1.2050	(1.1699, 1.2412)	1.1719	(1.1413, 1.2033)

### The causal mediation analysis for direct effect of sex on SICH prognosis

**Figure 1:**
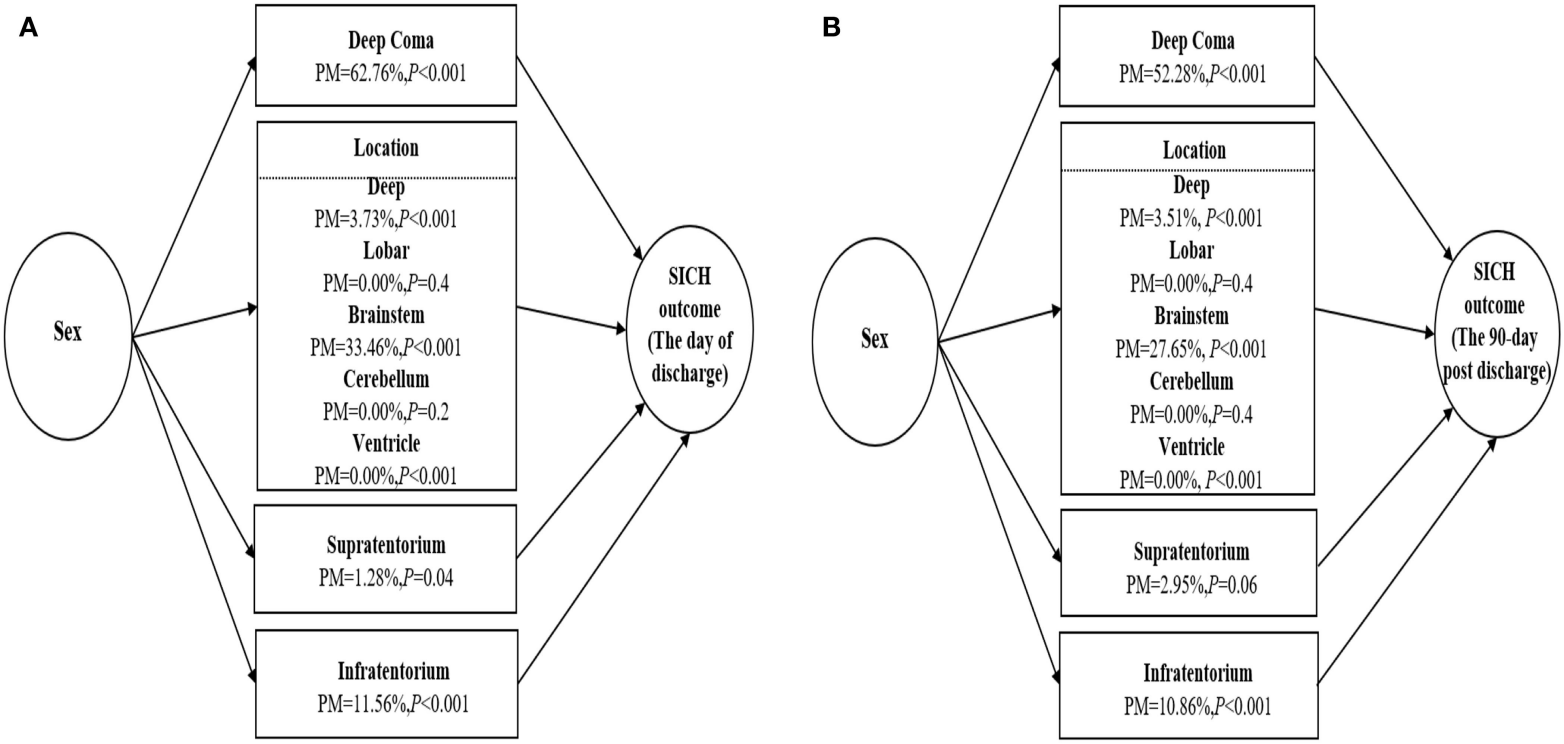
**(A)** Causal mediation analysis for sex association with spontaneous intracerebral hemorrhage (SICH) outcome on the day of discharge. **(B)** Causal mediation analysis for sex association with SICH outcome at the 90-day post-discharge. PM, proportion mediated.

After estimating the overall effects of sex on SICH outcomes on the day of discharge and the 90-day post-discharge, we performed a causal mediation analysis. This analysis allowed us to further decompose the overall effect into direct and indirect effects, and to estimate the average causal mediation effect for each mediator. As shown in Figure 1, the causal mediation analysis results were highly consistent, regardless of whether SICH outcome was measured on the day of discharge or at the 90-day post-discharge. Deep coma was the most likely mediator in the relationship between sex and SICH outcome, and accounted for ~50–60% of the effect of sex on SICH outcome. Brainstem hemorrhage was another mediator deserving of attention, with an average mediation effect of 20–30%. In addition, an infratentorial hemorrhage volume of >10 ml contributed ~10% to the effect of sex on the SICH outcome, and this could be examined further in future mechanical pathway studies.

## Discussion

This study calculated the effect of sex on SICH outcomes using the largest sample of real-world data to date, which guarantees representativeness and statistical power (15). Our results revealed that female patients had better prognostic outcomes than male patients. Moreover, this prognostic difference between the sexes attenuates with increasing age. Namely, for patients aged ≥75 years, there was no protective effect of the female sex. Causal mediation analysis revealed that the association between sex and SICH outcomes was probably mediated by the male frequency of deep coma, brainstem hemorrhage, and an infratentorial intracerebral hemorrhage volume of >10 ml.

Deep coma was found to play a key role in the relationship between sex and SICH outcomes. We found that patients with brainstem hemorrhage or an infratentorial intracerebral hemorrhage volume of >10 ml frequently suffered from a deep coma. The brainstem reticular formation has been considered essential for wakefulness, which can explain why deep coma is more commonly a result of brainstem hemorrhage than of hemorrhage in other brain areas (16–18). Additionally, given the narrow confines of the posterior fossa, a hernia can appear quickly in cerebellar hemorrhage with obstructive hydrocephalus (19). Consequently, significant infratentorial intracerebral hematoma could cause a deep coma through the hernia. Brainstem and cerebellum hemorrhage usually take place in small non-branching perforating arteries that have a diameter of 50–200 μm (20), which branch directly from larger arteries. According to a study, microatheroma is likely to form in these small arteries due to endothelial injury (20). Hence, endothelium injury could explain the sex-related difference in SICH prognosis found in the current study.

It is complicated to determine sex-related differences in endothelium injury, for which there are several potential biological pathways. One plausible explanation for the sex-related protection effect in SICH is that women's female gonadal hormones affect the endothelium. The endothelium-protective effects of estrogen could occur through various pathways, such as regulation of MAPK/PI3K/AKT signaling pathways, a decrease in prostaglandin E2 and cyclooxygenase 2 to reduce the inflammatory response, and modulation of nitric oxide synthase to reduce oxidative stress (21–24). Furthermore, the alteration of hormone levels during menopause has been associated with the incidence and outcomes of ischemic stroke and SICH. Although more than half of the female patients in the present study were postmenopausal, we cannot determine whether female gonadal hormones (especially estrogen) caused the sex-related differences in SICH prognosis. Nonetheless, we suspect that female gonadal hormones militate to some extent. Specifically, we found that the effects of sex on prognosis attenuated with increasing age. Moreover, the age-stratified analysis revealed that the sex-related difference in SICH prognosis was limited to patients younger than 75 years, and this tendency is in accordance with an endothelium protective effect of estrogen. Animal experiments have revealed that the administration of estrogen and progesterone improves ICH outcomes. However, hormone replacement therapy has been reported to make limited contributions to prognosis. Various aspects of animal experiments and clinical trials could account for the conflicting results, such as duration of exposure, dose, and administration route (24). With a limited understanding of female gonadal hormones, it is difficult to successfully perform hormone replacement therapy. Additionally, Gibson found that both short-term and long-term estrogen deficiency reduces the expression of estrogen receptors (25). This suggests that SICH in male patients could be improved through drugs that interact with estrogen receptors. Thus, future studies should investigate how female gonadal hormones protect the function of the endothelium, as well as the consequence of hormones in the pathogenesis and prognosis of SICH. The present findings could therefore assist the development of more effective therapies for patients with SICH. Additionally, our results hold significance for the prevention and prognosis prediction of ICH in both men and women and could support the formulation of community health policies.

### Strengths and limitations

This study has several strengths. First, our study included data from the largest sample size, which ensured statistical representativeness. Then, we used causal inference to explore the effects of sex on SICH prognosis, and adjusted for confounders to reduce bias, which revealed a widespread effect of sex on the SICH outcome. Finally, we proposed possible mediators for the relationship between sex and SICH prognosis, as well as the corresponding pathways, based on what is known in the field of neurology.

This study has some limitations that should be noted. First, we did not examine the effect of intracerebral hemorrhage expansion, even though this is an essential factor for the prognosis of SICH. The heterogeneity of the SICH expansion measurement method obstructed an analysis of the effects of hematoma expansion. However, results not reported here showed that the estimated effects of sex on SICH outcomes were robust when ICH expansion was included in the model. Second, we proposed some possible mediators in the relationship between sex and SICH outcomes, but clear insights on this have yet to be obtained and some questions remain. For example, how do these mediators perform in the pathway? Do they act independently or jointly? Are there interaction effects? Future studies should address these questions and this, together with our results, could shed light on sex-related biological pathways in SICH.

## Conclusion

We found that male patients had a higher risk of a poor SICH prognosis than female patients, and this was partially associated with deep coma, brainstem hemorrhage, and an infratentorial hemorrhage volume of >10 ml. It is necessary to further explore the biological mechanisms underlying the sex-related differences in SICH prognosis, which could facilitate the development of individual-based treatment.

## Data availability statement

The data analyzed in this study is subject to the following restrictions. Requests to access these datasets should be directed to the corresponding author XW, yuxixi1052006@126.com.

## Author contributions

TZ and JZ contributed to research design, data analysis, and article writing. HW and YY contributed to data analysis and article writing. JW and XW contributed to research design, as shown in Appendix e-1. All authors contributed to the article and approved the submitted version.

## Funding

This research work was funded by the Sichuan Science and Technology Program (grant numbers 2020YFS0091 and 2021YFS0001-LH), the Health Commission of Sichuan province (grant number 20PJ092), the Chongqing Science and Technology Program (grant number cstc2020jscx-cylhX0003), the Central Government funding items (grant numbers 2021zc02), the Chengdu Science and Technology Program (grant number 2021-YF05-01585-SN), and 1.3.5 project for disciplines of excellence, West China Hospital, Sichuan University (No. 2018HXFH010). The funders played no role in the design of the study and collection, analysis, and interpretation of data, and in the writing the manuscript.

## Conflict of interest

The authors declare that the research was conducted in the absence of any commercial or financial relationships that could be construed as a potential conflict of interest.

## Publisher's note

All claims expressed in this article are solely those of the authors and do not necessarily represent those of their affiliated organizations, or those of the publisher, the editors and the reviewers. Any product that may be evaluated in this article, or claim that may be made by its manufacturer, is not guaranteed or endorsed by the publisher.

## Appendix e-1

**Table A1:**

**Name**	**Locations**	**Roles**	**Contributions**
Tao Zhang	West China School of Public Health, Sichuan University, Chengdu, China	Research design, data analysis, and article writing	Research design, data analysis, and article writing
Jieyi Zhao	Department of Neurosurgery, West China Hospital, Chengdu, China	Research design, data analysis, and article writing	Research design, data analysis, and article writing
Hongli Wan	West China School of Public Health, Sichuan University, Chengdu, China	Data analysis and article writing	Data analysis and article writing
Yang Yu	Department of Neurosurgery, West China Hospital, Chengdu, China	Data analysis and article writing	Data analysis and article writing
Jin Wen	Institute of Hospital Management, West China Hospital, Sichuan University, Chengdu, China	Research design	Research design
Xiaoyu Wang	Department of Neurosurgery, West China Hospital, Chengdu, China	Research design	Research design

## References

[1] Spagnolo PA Manson JE Joffe H. Sex and gender differences in health: what the COVID-19 pandemic can teach us. Ann Intern Med. (2020) 173:385–6. 10.7326/M20-1941 32384135 PMC7249504

[2] Zhou J Zhang Y Arima H Zhao Y Zhao H Zheng D. Sex differences in clinical characteristics and outcomes after intracerebral haemorrhage: results from a 12-month prospective stroke registry in Nanjing, China. BMC Neurol. (2014) 14:172. 10.1186/s12883-014-0172-5 25182069 PMC4159550

[3] Gokhale S Caplan LR James ML. Sex differences in incidence, pathophysiology, and outcome of primary intracerebral hemorrhage. Stroke. (2015) 46:886–92. 10.1161/STROKEAHA.114.007682 25657181

[4] Marini S Morotti A Ayres AM Crawford K Kourkoulis CE Lena UK. Sex differences in intracerebral hemorrhage expansion and mortality. J Neurol Sci. (2017) 379:112–6. 10.1016/j.jns.2017.05.057 28716219 PMC5538146

[5] Roquer J Rodríguez-Campello A Jiménez-Conde J Cuadrado-Godia E Giralt-Steinhauer E Hidalgo RM. Sex-related differences in primary intracerebral hemorrhage. Neurology. (2016) 87:257–62. 10.1212/WNL.0000000000002792 27281527 PMC4955279

[6] Appelros P Stegmayr B Terent A. Sex differences in stroke epidemiology: a systematic review. Stroke. (2009) 40:1082–90. 10.1161/STROKEAHA.108.540781 19211488

[7] Zia E Engstrom G Svensson PJ Norrving B Pessah-Rasmussen H. Three-year survival and stroke recurrence rates in patients with primary intracerebral hemorrhage. Stroke. (2009) 40:3567–73. 10.1161/STROKEAHA.109.556324 19729603

[8] van Asch CJ Luitse MJ Rinkel GJ van der Tweel I Algra A Klijn CJ. Incidence, case fatality, and functional outcome of intracerebral haemorrhage over time, according to age, sex, and ethnic origin: a systematic review and meta-analysis. Lancet Neurol. (2010) 9:167–76. 10.1016/S1474-4422(09)70340-0 20056489

[9] Phan HT Reeves MJ Blizzard CL Thrift AG Cadilhac DA Sturm J. Sex differences in severity of stroke in the INSTRUCT study: a meta-analysis of individual participant data. J Am Heart Assoc. (2019) 8:e010235. 10.1161/JAHA.118.010235 30590965 PMC6405721

[10] Ganti L Jain A Yerragondu N Jain M Bellolio MF Gilmore RM. Female gender remains an independent risk factor for poor outcome after acute nontraumatic intracerebral hemorrhage. Neurol Res Int. (2013) 2013:219097. 10.1155/2013/219097 24083025 PMC3777128

[11] Umeano O Phillips-Bute B Hailey CE Sun W Gray MC Roulhac-Wilson B. Gender and age interact to affect early outcome after intracerebral hemorrhage. PLoS ONE. (2013) 8:e81664. 10.1371/journal.pone.0081664 24312335 PMC3842307

[12] Yesilot NF Koyuncu BA Coban O Tuncay R Bahar SZ. Gender differences in acute stroke: Istanbul medical school stroke registry. Neurol India. (2011) 59:174–9. 10.4103/0028-3886.79130 21483112

[13] Sheikh K Bullock CM. Effect of measurement on sex difference in stroke mortality. Stroke. (2007) 38:1085–7. 10.1161/01.STR.0000258103.15708.58 17255545

[14] James ML Cox M Xian Y Smith EE Bhatt DL Schulte PJ. Sex and age interactions and differences in outcomes after intracerebral hemorrhage. J Womens Health. (2017) 26:380–8. 10.1089/jwh.2016.5849 27754758

[15] Levenson MS. Regulatory-grade clinical trial design using real-world data. Clin Trials. (2020) 17:377–82. 10.1177/1740774520905576 32063037 PMC8697198

[16] Fischer DB Boes AD Demertzi A Evrard HC Laureys S Edlow BL. A human brain network derived from coma-causing brainstem lesions. Neurology. (2016) 87:2427–34. 10.1212/WNL.0000000000003404 27815400 PMC5177681

[17] Takeuchi S Suzuki G Takasato Y Masaoka H Hayakawa T Otani N. Prognostic factors in patients with primary brainstem hemorrhage. Clin Neurol Neurosurg. (2013) 115:732–5. 10.1016/j.clineuro.2012.08.022 22944466

[18] Fan Z Hao L Chuanyuan T Jun Z Xin H Sen L. Neutrophil and platelet to lymphocyte ratios in associating with blood glucose admission predict the functional outcomes of patients with primary brainstem hemorrhage. World Neurosurg. (2018) 116:e100–7. 10.1016/j.wneu.2018.04.089 29689388

[19] Hemphill III JC Greenberg SM Anderson CS Becker K Bendok BR Cushman M. Guidelines for the management of spontaneous intracerebral hemorrhage: A Guideline for Healthcare Professionals From the American Heart Association/American Stroke Association. Stroke. (2015) 46:2032–60. 10.1161/STR.0000000000000069 26022637

[20] Schrag M Kirshner H. Management of intracerebral hemorrhage: JACC focus seminar. J Am Coll Cardiol. (2020) 75:1819–31. 10.1016/j.jacc.2019.10.066 32299594

[21] Simon JA Hsia J Cauley JA Richards C Harris F Fong J. Postmenopausal hormone therapy and risk of stroke: The Heart and Estrogen-progestin Replacement Study (HERS). Circulation. (2001) 103:638–42. 10.1161/01.CIR.103.5.638 11156873

[22] Viscoli CM Brass LM Kernan WN Sarrel PM Suissa S Horwitz RI. clinical trial of estrogen-replacement therapy after ischemic stroke. N Engl J Med. (2001) 345:1243–9. 10.1056/NEJMoa010534 11680444

[23] Grady D Herrington D Bittner V Blumenthal R Davidson M Hlatky M. Cardiovascular disease outcomes during 68 years of hormone therapy: Heart and Estrogen/progestin Replacement Study follow-up (HERS II). JAMA. (2002) 288:49–57. 10.1001/jama.288.1.49 12090862

[24] Rossouw JE Anderson GL Prentice RL LaCroix AZ Kooperberg C Stefanick ML. Risks and benefits of estrogen plus progestin in healthy postmenopausal women: principal results From the Women's Health Initiative randomized controlled trial. JAMA. (2002) 288:321–33. 10.1001/jama.288.3.321 12117397

[25] Nakamura T Hua Y Keep RF Park JW Xi G Hoff JT. Estrogen therapy for experimental intracerebral hemorrhage in rats. J Neurosurg. (2005) 103:97–103. 10.3171/jns.2005.103.1.0097 16121980

